# Association of total daily physical activity with disability in community-dwelling older persons: a prospective cohort study

**DOI:** 10.1186/1471-2318-12-63

**Published:** 2012-10-16

**Authors:** Raj C Shah, Aron S Buchman, Sue Leurgans, Patricia A Boyle, David A Bennett

**Affiliations:** 1 Rush Alzheimer’s Disease Center, Rush University Medical Center, Chicago, IL, USA; 2 Department of Family Medicine, Rush University Medical Center, Chicago, IL, USA; 3 Department of Neurological Sciences, Rush University Medical Center, Chicago, IL, USA; 4 Department of Behavioral Sciences, Rush University Medical Center, Chicago, IL, USA

**Keywords:** Total daily physical activity, Disability, Activities of daily living, Actigraphy, Elderly, Longitudinal

## Abstract

**Background:**

Based on findings primarily using self-report measures, physical activity has been recommended to reduce disability in old age. Collecting objective measures of total daily physical activity in community-dwelling older adults is uncommon, but might enhance the understanding of the relationship of physical activity and disability. We examined whether greater total daily physical activity was associated with less report of disability in the elderly.

**Methods:**

Data were from the Rush Memory and Aging Project, a longitudinal prospective cohort study of common, age-related, chronic conditions. Total daily physical activity was measured in community-dwelling participants with an average age of 82 using actigraphy for approximately 9 days. Disability was measured via self-reported basic activities of daily living (ADL). The odds ratio and 95% Confidence Interval (CI) were determined for the baseline association of total daily physical activity and ADL disability using a logistic regression model adjusted for age, education level, gender and self-report physical activity. In participants without initial report of ADL disability, the hazard ratio and 95% CI were determined for the relationship of baseline total daily physical activity and the development of ADL disability using a discrete time Cox proportional hazard model adjusted for demographics and self-report physical activity.

**Results:**

In 870 participants, the mean total daily physical activity was 2. 9 × 10^5^ counts/day (range in 10^5^ counts/day = 0.16, 13. 6) and the mean hours/week of self-reported physical activity was 3.2 (SD = 3.6). At baseline, 718 (82.5%) participants reported being independent in all ADLs. At baseline, total daily physical activity was protective against disability (OR per 10^5^ counts/day difference = 0.55; 95% CI = 0.47, 0.65). Of the participants without baseline disability, 584 were followed for 3.4 years on average. Each 10^5^ counts/day additional total daily physical activity was associated with reduced hazard of developing disability by 25% (HR = 0.75, 95% CI = 0.66, 0.84). The results were unchanged after controlling for important covariates including cognition, depressive symptoms, and chronic health conditions.

**Conclusions:**

Greater total daily physical activity is independently associated with less disability even after controlling for self-reported physical activity.

## Background

Maintaining functional independence with aging is an important public health priority. In Healthy People 2020 [1], reduction by 10-percentage points of older Americans reporting moderate to severe functional limitation over the next decade is listed as a national goal. For this goal, moderate to severe functional limitation is defined as reporting difficulty in performing at least one basic activity of daily living (ADL) [2]. While almost 30% of adults 65 years and older reported functional dependence, the prevalence increases from about 20% in persons between 65 and 74 to over 55% in persons over age 85 [3]. During the last few decades, the prevalence of functional independence in older persons has increased [4,5]. As more Americans are living into their eighties, though, the absolute number of older Americans reporting functional limitations may increase [6].

Using the best available scientific evidence [7,8], public health messages from the Centers for Disease Control [9] and the National Institute on Aging [10] encourage older persons to increase physical activity levels in order to maintain functional independence. However, there are some important gaps in the scientific underpinnings of these public health messages. First, there is a paucity of longitudinal studies examining the association of physical activity and development of disability in community-dwelling persons over the age of 80 [11]. Second, a recent critical appraisal [12] highlighted inconsistencies in the evidence regarding increased late-life physical activity and disability. Most [13-15], but not all [16], longitudinal studies of community-dwelling, older adults have shown an association. One reason that these studies may have produced mixed results is the use of self-report measures of physical activity. In older persons, self-report measures may be affected by recall bias due to cognitive function changes and may not capture the full extent of movement throughout the course of a day.

Due to the potential limitations of self-report physical activity in community-dwelling cohorts over the age of 80, researchers have focused on utilizing performance-based measures. Portable actigraphy offers the recording, storage, and analysis of motion measured by accelerometers in community-dwelling persons. As a result, objective measures of total daily physical activity over the course of a week or more can be determined in a cost-effective and efficient manner [17]. While actigraphy has been applied in population studies of physical activity in younger adults [18,19], these studies have not included participants over the age of 80.

In the Rush Memory and Aging Project [20], an ongoing, longitudinal investigation of common chronic conditions of old age, portable actigraphy data were collected in a large group of community-based older persons with an average age over 80 years. Total daily physical activity, which includes both exercise and non-exercise activities, was quantified. In addition, disability evaluations were conducted annually for up to five years. Persons reporting any limitations on any of the ADLs were deemed to have disability. We tested the hypotheses that greater total daily physical activity was associated with less prevalent disability and with a lower incidence of ADL disability. As prior work in this cohort showed that greater self-report of hours of physical activity per week was associated with a lower hazard of developing ADL disability in the future [21], we also examined if greater total daily physical activity still was associated with low probability of report of ADL disability after controlling for self-report physical activity.

## Methods

### Participants

All participants were older, community-dwelling individuals in the Rush Memory and Aging Project [20]. Participants were recruited from about 40 continuing care retirement communities in the Chicago, Illinois vicinity along with churches and social service agencies (see “Acknowledgments”). Participants agreed to annual clinical evaluations and brain donation at the time of death. The study was approved by the Institutional Review Board of Rush University Medical Center. Written informed consent was obtained from all study participants.

The Rush Memory and Aging Project began in 1997. The study is still ongoing with rolling enrolment. As of January 2011, 1332 participants (75.6% living in continuing care retirement communities and the remainder in private residences) had completed an initial evaluation, of which 892 were enrolled following addition of actigraphy 5.2 years earlier. Of participants with actigraphy, 870 (97.5%) had a concurrent assessment of disability. In these analyses, the first evaluation with an assessment of functional independence along with actigraphy data was defined as baseline.

### Assessment of disability

A modified version of the Katz Index was used to measure independence with basic ADLs [22]. Participants reported whether they needed (1) no help, (2) help, or (3) were unable to do each of six activities: bathing, dressing, feeding, toileting, transferring, and walking across a small room. The number of activities participants reported as needing help or unable to perform was calculated for each evaluation. Disability was defined as being dependent in at least one basic ADL.

### Quantitative total daily physical activity

All Rush Memory and Aging Project participants were eligible to participate in the collection of actigraphy data as long as they agreed to wearing an Actical^©^ (Mini Mitter, Bend, OR), a device with a piezo-electric accelerometer. Participants were instructed to wear the actigraphy device continuously without taking it off. The non-dominant wrist was chosen as the application site to facilitate participants’ ability to wear the device throughout the measurement period. Actical^©^ retrieval was scheduled for 10 days later but could vary depending on participant availability. Upon retrieval of the actigraph, raw data were downloaded and viewed using software provided by Respironics, Inc (Bend, OR) to confirm if recording failures had occurred. Data then were partitioned into 24 hour periods from the time of placement to the time of retrieval, and only data from complete 24 hours periods were used to determine average total daily physical activity. Actical^©^ measures do not directly correspond to observed movements but are proportional to the degree and intensity of movements as reflected in recorded activity curves. Activity counts represent the area calculated by integrating the activity curve for each 1-second sample, where non-zero values reflected activity. Activity counts are summed for each epoch (15 second). Total daily physical activity was the mean sum of all 24-hour activity counts [23].

### Self-report physical activity

Using questions adapted from the 1985 National Health Interview Survey, self-report physical activity was assessed [24]. Participants were asked if they had engaged in any of five activities (exercise, gardening or yard work, calisthenics or general exercise, bicycle riding, and swimming or water exercise) within the previous two weeks. Using the number of occasions for each reported activity per week and the average number of minutes per occasion, the minutes spent engaged in each activity were summed and divided by 120 to yield a composite measure of participation in physical activity expressed as hours per week [25].

### Covariates

Participants were asked for demographic information including date of birth, sex, and highest number of years of education completed. Other covariates included global cognitive function, dementia diagnosis, vascular risk factors and vascular disease burden, joint pain, body mass index, and depressive symptoms. Cognitive function was assessed using a battery of 19 tests which were used to create a composite measure of global cognition as described elsewhere [20]. Dementia was diagnosed in a three-step process. Cognitive testing was scored by computer and reviewed by a neuropsychologist to diagnose cognitive impairment. Participants then were evaluated by a clinician who used all cognitive and clinical data to diagnose AD using the NINDS-ADRDA criteria [26]. Utilizing self-report questions, clinical examination, and medication inspection, summary scores for each individual’s vascular risk burden (i.e., the number out of the 3 risk factors diabetes mellitus, hypertension, and smoking reported) and vascular disease burden (i.e., the number out of the 4 vascular diagnoses claudication, congestive heart failure, heart attack, and stroke) were computed, as previously described [27]. Participants were asked whether they had experienced persistent joint pain in the previous month [28]. Body mass index was calculated by dividing measured weight in kilograms by measured height in meters squared [29]. Number of depressive symptoms experienced in the prior week was assessed using a 10-item version of the Center for Epidemiologic Studies Depression Scale [30].

### Statistical analysis

Statistical programming was done in SAS, Version 9.2 (SAS Institute, Inc., Cary, NC). t-tests (with Satterthwaite adjustments for unequal variances, when appropriate) and nonparametric tests (as appropriate) were used to compare the baseline characteristics of participants with total daily physical activity. Then, the cross-sectional association between total daily physical activity and prevalent disability at baseline was examined using a logistic regression model adjusted for age, education, sex and subsequently was repeated by limiting the cohort to participants age 80 and older at baseline. Then, the initial model was repeated five times with the addition of terms for global cognitive function, vascular risk factors and vascular disease, joint pain, body mass index, and depressive symptoms. Next, the initial model was repeated with a term for self-reported physical activity in addition to a term for total daily physical activity. In order to examine the relationship with total daily physical activity and incident disability, discrete time proportional hazards models [31] of the time to first report of disability were adjusted for age, sex, and education and then repeated by limiting the cohort to participants age 80 and older at baseline. Subsequent models added the demographic and clinical covariates in the order described for the logistic regression models. Finally, the initial proportional hazards model was repeated after excluding participants with a diagnosis of dementia at baseline. Given multiple comparisons, significant p-values were set at a value of 0.01.

## Results

### Baseline participant characteristics

There were 870 participants with valid concurrent Actical^©^ and ADL measures (mean age 81.9 years (SD = 7.2), 73% women, and mean education 14.8 years (SD = 3.0)). Of the 870 participants, 569 (65%) were greater than or equal to 80 years of age and 155 (18%) had a self-report of physical activity equal to zero hours/week. Total daily physical activity counts/day were based on readings from a mean of 9.3 days (SD = 1.1). Total daily physical activity ranged widely from 0.16 × 10^5^ counts/day to 13.6 ×10^5^ counts/day (mean = 2.9 × 10^5^ counts/day; SD = 1.6 × 10^5^ counts/day). The distribution of total daily physical activity was positively skewed (skewness = 1.2). At baseline, higher total daily physical activity was associated with younger age, a lower total score for report of ADL disability, more self-reported hours of physical activity per week, better cognitive performance, fewer reports of vascular disease risk factors, fewer reports of vascular diseases, and fewer depressive symptoms (Table 1).

**Table 1:** Participant characteristics and correlation with total daily physical activity

**Baseline characteristic**	**Value** (**n = 870**)	**Spearman correlation with total daily physical activity**
Age, mean (SD), years	81.9 (7.3)	−0.26, p < 0.001
Women, Number (%)	349 (73.2)	−0.06, p = 0.104
Education, mean (SD), years	14.8 (3.0)	−0.03, p = 0.40
Activity of Daily Living score, mean (SD), out of 6	0.4 (1.1)	−0.28, p < 0.001
Reported Physical Activity, mean (SD), hours per week	3.20 (3.57)	0.25, p < 0.001
Global Cognition, mean (SD), z-score	0.05 (0.68)	0.23, p < 0.001
Number of vascular risk factors, mean (SD), out of 3	1.24 (0.83)	−0.12, p < 0.001
Number of vascular diseases, mean (SD), out of 4	0.47 (0.77)	−0.21, p < 0.001
Report of joint pain, Number (%)	168 (36.6%)	−0.06, p = 0.09
Body Mass Index, mean (SD), kg/m^2	27.0 (5.3)	−0.08, p = 0.03
Depressive symptoms, mean (SD), out of 10	1.18 (1.75)	−0.13, p < 0.001

SD=standard deviation.

In order to confirm that total daily physical activity did not completely overlap with ADL disability report, we determined the correlation coefficient of the two variables in persons with self-report of no physical activity (n = 155). The mean level of total daily physical activity for persons reporting no physical activity was 2.5 × 10^5^ (SD = 1.5) counts/day. The correlation between each 10^5^ activity counts/day in total daily physical activity and total score on the ADL disability scale was 15%, showing some but not complete overlap.

### Total daily physical activity and prevalent disability

Of the 870 participants with baseline actigraphy and concurrent ADL assessment, 718 (82.5%) reported functional independence. In a logistic regression model adjusted for age, education, and sex, with the report of dependence on any ADL as the outcome, the odds ratio for each additional 10^5^ counts/day of total daily physical activity was 0.55 (95% CI = 0.47, 0.65), so that greater total daily physical activity was associated with lower likelihood of reporting disability. When limiting the primary analysis to the 569 participants age 80 or over at baseline, the odds ratio for prevalent disability per 10^5^ activity/counts per day was 0.50 (95% CI = 0.41, 0.62). The association for total daily activity and disability was unchanged when adding global cognitive function (OR = 0.61; 95% CI = 0.52, 0.72). The association was not affected by the presence of vascular risk factors and diseases, body mass index, joint pain, and depressive symptoms (data not shown). Then, the initial model was repeated adding a variable representing hours/week of physical activity to determine if total daily activity provided additional information to self-report physical activity. Greater activity with each measure was associated with lower likelihood of disability (OR per 10^5^ counts/day difference in total daily activity = 0.60, 95% CI = 0.50, 0.70; OR per hour/week difference in self-reported physical activity = 0.89; 95% CI = 0.82, 0.96).

### Total daily physical activity and incident disability

To examine the association between total daily physical activity and the development of disability, we looked at all persons at risk for incident disability who were followed longitudinally. Of the 718 participants without baseline disability, 134 did not have at least one annual follow-up evaluation of disability (12 died, 107 had not reached their initial follow-up visit at the time of these analyses, and 15 did not have an additional measure of ADL on follow-up) and were excluded from the longitudinal analyses. Of the remaining 584 participants (81.3% of 718), 182 persons (31.2% of 584) reported dependence for at least one ADL during the course of follow-up. Baseline characteristics between participants developing disability and participants not developing disability are compared in Table 2 and 66% of the participants were 80 years of age or older. Total daily physical activity counts/day were based on readings from a mean of 9.2 days (SD = 1.1). Mean total daily physical activity of the 584 participants was 3.1 × 10^5^ counts/day (SD = 1.5 × 10^5^ counts/day). Over the course of a maximum 5.2 years of follow-up, participants underwent a mean of 3.4 (SD = 1.3) annual follow-up evaluations.

**Table 2:** Participant characteristics by incident disability*

**Baseline characteristic**	**Developed disability**		**p**-value**
	**Yes**	**No**	
	(**n = 182**)	(**n = 402**)	
Age, mean (SD), years	84.1 (5.7);	80.7 (7.2);	t_581_= −5.75, p < 0.001
Women, Number (%)	147 (80.8)	290 (72.1)	*χ*_1_^2^= 4.8, p = 0.03
Education, mean (SD), years	14.2 (2.8)	15.0 (2.9)	t_581_= 3.33, p < 0.001
Total Daily Physical Activity, mean (SD), 10^5^ counts/day	2.7 (1.4)	3.2 (1.5)	t_581_= 4.07, p < 0.001
Reported Physical Activity, mean (SD), hours per week	2.49 (3.14)	3.69 (3.83)	t_579_= 3.68, p < 0.001
Global Cognition, mean (SD), z-score	−0.13 (0.69)	0.22 (0.61)	t_580_= 6.15, p < 0.001
Number of vascular risk factors, mean (SD), out of 3	1.21 (0.78)	1.18 (0.82)	t_580_= −0.35, p = 0.7
Number of vascular diseases, mean (SD), out of 4	0.54 (0.80)	0.38 (0.67)	t_580_= −2.58, p = 0.01
Report of joint pain, Number (%)	78 (42.9%)	120 (29.9%)	*χ*_1_^2^= 9.63, p = 0.002
Body Mass Index, mean (SD), kg/m^2	27.6 (5.7)	26.3 (4.6)	t_530_= −2.80, p = 0.005
Depressive symptoms, mean (SD), out of 10	1.43 (1.75)	0.88 (1.52)	t_580_= −3.79, p < 0.001
Follow-Up Time, mean (SD), years	3.7 (1.0)	3.3 (1.3)	t_581_= −3.52, p < 0.001

* Disability defined by report of not being able to independently perform at least one basic activity of daily living.

** Statistical significance based on t tests or chi-square tests, as appropriate.

SD = standard deviation.

**Figure 1:**
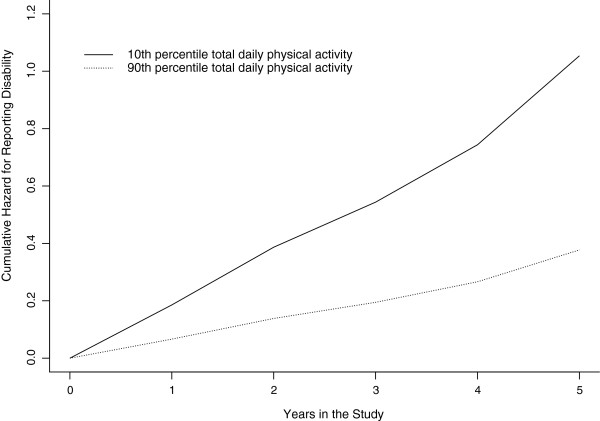
**Total daily physical activity and incident disability.** The cumulative hazard of developing an activities of daily living disability as a function of time in study from a discrete time proportional hazards model adjusted for age, sex, and education level for an average participant (82 year-old woman with 14.8 years of education) with a total daily physical activity level in the 10^th^ percentile (1.0 × 10^5^ counts/day) as depicted by the solid curve and for an average participant with a total daily physical activity level in the 90^th^ percentile (4.7 × 10^5^counts/day) as depicted by the dashed curve

In a discrete time proportional hazards model adjusted for age, sex, and education, the hazard of developing disability was 25% less for each additional 10^5^ counts/day of total daily physical activity (HR = 0.75, 95% CI = 0.66, 0.84). As depicted in Figure 1, the hazard of developing disability was 8.9 (95% CI = 7.6, 10.5) higher for a typical study participant (82 year-old woman with 14.8 years of education) who had low (10^th^ percentile or total daily physical activity of 1.0 × 10^5^ counts/day) when compared to a participant who had high total daily physical activity of (90^th^ percentile or total daily physical activity of 4.7 × 10^5^counts/day). When limiting the analysis to the 384 participants age 80 or over at baseline, the hazard ratio for developing disability was 0.78 (95% CI = 0.68, 0.90).

Then, we repeated the initial model controlling for baseline global cognitive function followed by four additional models controlling for vascular risk factors and vascular disease burden, joint pain, body mass index, and depressive symptoms. The association between total daily physical activity and development of disability was unchanged in all analyses (see Table 3). After excluding individuals with baseline dementia (n = 40), the hazard of developing disability for each additional 10^5^ counts/day of total daily physical activity was 0.74 (95% CI = 0.67, 0.88).

**Table 3:** Total daily physical activity and incident disability controlling for covariates*

	**Hazard for developing disability(95%CI)**				
**Variable**	**Model A**	**Model B**	**Model C**	**Model D**	**Model E**
Total daily physical activity	0.79(0.70,0.89)	0.75(0.67,0.85)	0.75(0.66,0.85)	0.76(0.67,0.86)	0.76(0.68,0.86)
Global Cognition	0.58(0.46,0.72)				
Vascular Risks		1.07(0.89,1.29)			
Vascular Disease		1.00(0.83,1.21)			
Joint Pain			1.59(1.18,2.14)		
Body Mass Index				1.06(1.03,1.09)	
Depressive Symptoms					1.14(1.06,1.24)

*From discrete time proportional hazard models adjusted for age, sex, and education where incident disability is measured as years to first report of not being independent in at least one basic activity of daily living.

Finally, we repeated our model controlling for self-report physical activity in order to determine if greater total daily physical activity continued to be independently associated with a lower hazard of developing disability. As shown in Table 4 (Model B), both total daily physical activity and self-report physical activity were independent predictors of lower hazard for report of incident disability.

**Table 4:** Relationship of total daily physical activity with incident disability*

	**Incident disability**	
	**Hazard ratio (95% Confidence interval)**	
**Variable**	**Model A**	**Model B**
Total daily physical activity	0.75 (0.66, 0.84)	0.77 (0.68, 0.87)
Self-report physical activity		0.94 (0.88, 0.99)

*From discrete time proportional hazard models adjusted for age, sex, and education where incident disability is measured as years to first report of not being independent in at least one basic activity of daily living.

## Discussion

In a large cohort of community-based older persons, greater total daily physical activity was associated with less report of disability at baseline and longitudinally, including in persons age 80 and older. The results were unchanged after adjusting for a wide variety of potential confounding variables including global cognitive function, vascular risk and disease burden, joint pain, body mass index, and depressive symptoms. In addition, the exclusion of persons with baseline dementia did not alter the results. Total daily physical activity remained associated with functional independence even after controlling for self-reported physical activities. These analyses support the link between total daily physical activity and disability and suggest an active lifestyle including high levels of not only exercise but also non-exercise physical activity may contribute to the maintenance of independence even in very old adults.

Our study of Rush Memory and Aging Project participants enhances the prior understanding of late-life physical activity and disability in three important ways. First, our study shows actigraphy measures can be obtained in community-dwelling older persons, including persons over age 80. As prior studies mainly reported the feasibility of actigraphy in children and middle-aged adults [18,19], extending the range to persons over the age of 80 points to actigraphy being a useful performance-based measure of activity throughout the lifespan.

Second, we also were able to show that total daily physical activity is a predictor of developing ADL disability, including persons aged 80 years and older. While prevention of ADL disability is a public health target for maintaining healthy populations, achieving a 10% reduction in disability in a decade [1] requires focused efforts on populations at high risk for developing disability such as persons over the age of 80. Public health efforts to encourage higher levels of total daily physical activity in this group may be useful especially since total daily physical activity remained a predictor even after controlling for other common age-related conditions associated with disability such as lower level of cognitive function. Prior work in the Rush Memory and Aging Project has highlighted the relationship between total daily physical activity and cognitive function [23], and cognitive decline is predictor of disability in older persons. Our study highlights that the association between total daily physical activity and disability is not completely explained by level of cognitive function and identifies the need for further studies to examine the mechanism by which greater total daily activity is associated with less disability.

Third, we were able to show that total daily physical activity and self-report physical activity provided separate and complementary predictive information for developing disability by measuring non-overlapping facets of physical activity in older adults. Why total daily physical activity and self-report physical activity provide complementary information requires further inquiry. Self-report of physical activity over an extended period may be highly influenced by level of physical activity in the last 24 hours and may capture intermittent intensity of physical activity. Total daily physical activity assessed continuously over multiple days incorporates all physical activity and may capture the mean levels for physical activity over the entire measurement period. Accumulating evidence in young and middle age individuals suggests that any movement (goal-directed or non-goal directed) may have important health outcomes [32] by increasing total daily energy expenditure [33]. The results of this study extend the prior findings in younger persons to the oldest of the old. Older persons, for whom participation in formal exercise may be constrained because of underlying health problems, may nonetheless benefit from increases in their levels of non-exercise physical activities.

Given that actigraphy is inexpensive and portable, total daily physical activity may be a useful method for clinically titrating physical activity interventions in older adults at risk for developing ADL disability. In our analysis, about 1 in 6 older persons without initial disability had no self-reported physical activity and their mean total daily physical activity level was 2.5 × 10^5^ counts/day. This information can be used to define a clinically useful two-step screening and intervention algorithm. In the first step, self-report physical activity may be utilized to quickly screen older adults without disability but at higher risk for developing disability. As self-report physical activity has limitations including recall bias, older persons with no self-report physical activity then can undergo measurement of total daily physical activity. If total daily physical activity is less than 2.5 × 10^5^ counts/day, more efforts can be made to encourage increasing total daily physical activity. Based on the current study findings, an increase in level of total daily physical activity from 2.5 × 10^5^ counts/day to 3.0 × 10^5^ counts/day (50^th^ percentile) could potentially result in a 14% reduction in hazard of developing ADL disability. Whether an increased in total daily physical activity is achieved can be monitored using actigraphy. While possible, such an evaluation and intervention algorithm in clinical settings requires further validation.

This study has several strengths. First, concomitant measures of total daily physical activity by actigraphy and a self-report measure of physical activity were available in a large cohort of community-based persons with a high rate of follow-up participation. Second, detailed cognitive function data enabled us to examine the effect of global cognition and dementia on the relation between physical activity and disability. Third, we were able to examine the association of total daily physical activity and disability in an older community-dwelling cohort. This study also has limitations including the use of a volunteer cohort. Second, total daily physical activity as measured by actigraphy does not classify the types of activities performed. Finally, the high education level of participants may limit the generalizability of the results to persons with less than 12 years of formal schooling.

## Conclusions

To our knowledge, there are no prior longitudinal studies using actigraphy as a performance-based method of assessing the relationship between late-life physical activity and disability in community-dwelling older persons. Our study points to performance-based assessment of total daily activity adding value by providing information complementary to self-report of physical activity while not being affected by recall biases associated with self-report in older persons at risk for cognitive decline. In the public health effort to delay disability in old age, total daily physical activity may be an important factor. Emphasis on a more active lifestyle with non-exercise activities along with exercise activities in public health messaging to persons over age 80 may be important, especially if our study results are replicated in other ongoing longitudinal cohort studies of older adults [17].

## Competing interests

The authors declare that they have no competing interests.

## Authors’ contributions

RCS and ASB conceived of the study, participated in its design and coordination, and drafted the manuscript. SL performed the statistical analysis and provided critical content revision of the manuscript. PAB and DAB contributed to the analysis and interpretation of the data, and provided critical content revision of the manuscript. All authors read and approved the final manuscript. RCS had full access to all of the data in the study and takes responsibility for the integrity of the data and the accuracy of the data analysis.

## Pre-publication history

The pre-publication history for this paper can be accessed here:

http://www.biomedcentral.com/1471-2318/12/63/prepub
